# Recombinant protein production in the new Millennium

**DOI:** 10.1186/1475-2859-6-33

**Published:** 2007-10-20

**Authors:** Antonio Villaverde, Diethard Mattanovich

**Affiliations:** 1 Institute for Biotechnology and Biomedicine, Autonomous University of Barcelona, Bellaterra, 08193 Barcelona, Spain; 2 Department of Genetics and Microbiology, Autonomous University of Barcelona, Bellaterra, 08193 Barcelona, Spain; 3 CIBER-BBN Networking Centre on Bioengineering, Biomaterials and Nanomedicine, Bellaterra, 08193 Barcelona, Spain; 4 University of Natural Resources and Applied Life Sciences Vienna, Department of Biotechnology, Institute of Applied Microbiology, Vienna, Austria; 5 School of Bioengineering, University of Applied Sciences FH-Campus Vienna, Austria

In 2000, the Microbial Physiology Section of the European Federation of Biotechnology gathered the academic and industrial community involved in the production of heterologous proteins for an update on physiological aspects governing protein production in prokaryotic and eukaryotic cells. While there was common understanding that the limits of efficiency in productivity and quality had by far not been reached, that meeting concentrated on a comparative description of physiological events, from genetic stability via transcription and translation to protein folding and secretion [1]. Despite the progressive comprehension of physiological limitations and cellular stresses in producing cells, there were only few examples of cell engineering towards improved expression systems.

In a biannual rhythm, the 4^th ^conference of this series was held in Barcelona in 2006, setting out to compile the achievements of novel genome wide analyses on the understanding and engineering of protein producing cell factories. Microbial Cell Factories provided then a forum for the publication of novel research presented at this conference, publishing all of the more than 100 abstracts presented in the conference [2] and selected papers. It became obvious that research in the field has made a major leap since 2000, from mere analytical approaches to complex and targeted cell engineering.

The development of a novel yeast vector was described in detail [3] and novel insight of plasmid/host interactions in *Escherichia coli *was presented [4]. At the transcript level, RNA stability was engineered in *E. coli *by downregulation of RNaseE [5], and novel regulatory mechanisms of transcription by estradiol in yeast were described [6]. While several authors studied heterologous protein secretion in a more general way [7,8], Resina et al. concentrated on induction of the unfolded protein response in yeasts [9]. As the production and secretion of many proteins is obviously still handicapped, there is still significant research on new, non-conventional expression systems, like psychrophilic bacteria [10], gram-positive bacteria [11] or fungi [3]. Being molecular physiology a key parameter for protein overexpression, one should not overlook the impact of process control. Maurer et al. have resolved a widely debated question – the dependence of specific productivity on cell growth – for protein secreting yeasts, and applied this information to predict an optimum feed rate profile for maximum productivity [12].

But future will not wait: the next Recombinant Protein Production conference is scheduled for 24^th^–28^th ^September, 2008, in Sardinia, Italy. In the way to explore cell physiology applied to improved protein production, successful cell engineering based on thorough – at best quantitative – understanding of the physiological limitations will be discussed: energy metabolism related to heterologous protein production, latest data on transcription and translation control, stress responses, protein folding and assembly, glycoengineering, and process development, stability and analytics in the light of host physiology. The Microbial Physiology Section of EFB will again welcome the Recombinant Protein Production community to a scientifically outstanding and stimulating meeting. More information will be found at the web site of the 5th Recombinant Protein Production Meeting [13].

## References

[B1] Merten O W, Mattanovich D, Cole J, Lang C, Larsson G, Neubauer P, Porro D, Postma P, Mattos JT (2001). Production of recombinant proteins with prokaryotic and eukaryotic cells.

[B2] (2006). The 4th Recombinant Protein Production Meeting: a comparative view on host physiology Barcelona, Spain. 21-23 September 2006. Abstracts. Microb Cell Fact.

[B3] Steinborn G, Boer E, Scholz A, Tag K, Kunze G, Gellissen G (2006). Application of a wide-range yeast vector (CoMed) system to recombinant protein production in dimorphic Arxula adeninivorans, methylotrophic Hansenula polymorpha and other yeasts. Microb Cell Fact.

[B4] Wang Z, Xiang L, Shao J, Wegrzyn A, Wegrzyn G (2006). Effects of the presence of ColE1 plasmid DNA in Escherichia coli on the host cell metabolism. Microb Cell Fact.

[B5] Kemmer C, Neubauer P (2006). Antisense RNA based down-regulation of RNaseE in E. coli. Microb Cell Fact.

[B6] Quintero MJ, Maya D, revalo-Rodriguez M, Cebolla A, Chavez S (2007). An improved system for estradiol-dependent regulation of gene expression in yeast. Microb Cell Fact.

[B7] Rodriguez AP, Leiro RF, Trillo MC, Cerdan ME, Siso MI, Becerra M (2006). Secretion and properties of a hybrid Kluyveromyces lactis-Aspergillus niger beta-galactosidase. Microb Cell Fact.

[B8] Surribas A, Resina D, Ferrer P, Valero F (2007). Rivoflavin may interfere with on-line monitoring of secreted green fluorescence protein fusion proteins in Pichia pastoris. Microb Cell Fact.

[B9] Resina D, Bollok M, Khatri NK, Valero F, Neubauer P, Ferrer P (2007). Transcriptional response of P. pastoris in fed-batch cultivations to Rhizopus oryzae lipase production reveals UPR induction. Microb Cell Fact.

[B10] Cusano AM, Parrilli E, Marino G, Tutino ML (2006). A novel genetic system for recombinant protein secretion in the Antarctic Pseudoalteromonas haloplanktis TAC125. Microb Cell Fact.

[B11] Yang Y, Biedendieck R, Wang W, Gamer M, Malten M, Jahn D, Deckwer WD (2006). High yield recombinant penicillin G amidase production and export into the growth medium using Bacillus megaterium. Microb Cell Fact.

[B12] Maurer M, Kuhleitner M, Gasser B, Mattanovich D (2006). Versatile modeling and optimization of fed batch processes for the production of secreted heterologous proteins with Pichia pastoris. Microb Cell Fact.

[B13] (2008). The 5th Recombinant Protein Production Meeting: a comparative view on host physiology. http://www.ing.univpm.it/rpp2008.

